# Tenascin-C expression and distribution in orthodontic treatment-induced gingival enlargement: A comparative immunohistochemical study

**DOI:** 10.4317/jced.64522

**Published:** 2026-09-15

**Authors:** Víctor Simancas-Escorcia, Ana Mercado-Perez, Martha Rebolledo-Cobos, Isaias Castilla-Castro, Antonio Díaz-Caballero, Adel Martínez-Martínez, Jaime Plazas-Román, Carlos M. Ardila

**Affiliations:** 1 Faculty of Health Sciences, GISACC Research Group, Corporación Universitaria Rafael Núñez, Cartagena 130001, Colombia; 2 Faculty of Health Sciences, SEIFO Research Group, Corporación Universitaria Rafael Núñez, Cartagena 130001, Colombia; 3 Faculty of Health Sciences, SICS Research Group, Fundación Universitaria San Martín, Puerto Colombia campus 081001, Atlántico, Colombia; 4 Faculty of Dentistry, GITOUC Research Group, Universidad de Cartagena, Cartagena 130015, Colombia; 5 Department of Periodontics, Saveetha Dental College, and Hospitals, Saveetha Institute of Medical and Technical Sciences, Saveetha University, Saveetha 600077, India; 6 Basic Sciences Department, Biomedical Stomatology Research Group, Faculty of Dentistry, Universidad de Antioquia U de A, Medellín 050010, Colombia

**Keywords:** Tenascin-C, gingival enlargement, orthodontics, orthodontic appliances, collagen.

## Abstract

**Background:**

Orthodontic treatment-induced gingival enlargement (OTGE) is the most common uncontrolled increase in gingival tissue in patients undergoing orthodontic treatment. The aim of this study was to identify the immunohistochemical expression and distribution of Tenascin-C in patients with OTGE.

**Materials and Methods:**

A descriptive observational study with non-probabilistic convenience sampling was conducted in 65 subjects. The control group comprised periodontally healthy individuals not undergoing orthodontic treatment (n=30), and the patient group comprised individuals diagnosed with generalized OTGE (Seymour index ≥30) (n=35). Immunohistochemical analysis was performed using a primary anti-Tenascin-C antibody (mouse monoclonal, clone 4C8MS, Novus Biologicals). Tenascin-C staining intensity was classified as 0 (absent), 1 (weak but detectable), 2 (moderate), and 3 (strong). Observations were made using a Leica DM 500 microscope.

**Results:**

Hypertrophic and hyperplastic epithelium was observed in 100% of OTGE patients. In the basal layer, keratinocytes did not maintain a parallel, continuous arrangement. The thin, continuous band of Tenascin-C was undetectable in 30 OTGE patients, while 5 patients showed weak but detectable expression. Weak immunohistochemical staining of blood vessels was found in 20 OTGE patients, and moderate staining in 15 patients.

**Conclusions:**

Compared with periodontally healthy controls, patients with OTGE showed altered immunohistochemical expression and distribution of Tenascin-C in the gingival basement membrane, fibroblasts, and blood vessels. These cross-sectional findings demonstrate an association between OTGE and altered Tenascin-C distribution.

## Introduction

Orthodontic treatment-induced gingival enlargement (OTGE), also known as gingival fibromatosis, hypertrophy, or hyperplasia, is the uncontrolled increase of the marginal gingiva, attached gingiva, or interdental papilla [1]. This pathological condition has been associated with systemic diseases and as an undesired effect of the prolonged intake of certain medications whose pharmacological target is not the gingival tissue (calcium channel blockers, immunosuppressants, and anticonvulsants) [2,3]. Clinical studies indicate that OTGE appears as a localized or generalized pathological entity in patients undergoing orthodontic treatment [4]. Although one of the aims of orthodontic treatment is to achieve dental harmonization according to physiological and esthetic criteria, research has shown that morphological changes in the gingiva of these patients can appear as early as the first month after treatment onset, with a prevalence of up to 55% in studied populations [5,6].

Several etiological factors have been associated with the onset of OTGE. These include bacterial biofilm deposition and inefficient removal, and mechanical and chemical irritation caused by dental cements and orthodontic bands [7,8]. OTGE has also been reported in patients with adequate oral hygiene and no signs of inflammation [9]. Mechanical stress induced by compressive or tensile forces on the periodontal tissue, as well as nickel accumulation in the gingival tissue of orthodontic patients, have also been related to the onset of this pathology [4,10]. It is likely that the anatomical changes induced by these etiological factors predispose orthodontic patients to periodontal disease and halitosis, and negatively affect oral health-related quality of life [11].

Excessive deposition of extracellular matrix (ECM) proteins or ground substance appears to be one of the histological characteristics of the gingival connective tissue in OTGE patients, including collagen fiber accumulation [12]. However, collagen deposition is likely only one essential part of the hyperplastic process. Therefore, the study of other ECM components, such as glycoproteins, is warranted. A glycoprotein of great interest in recent years due to its role in sites of high cell turnover, plasticity, and cell remodeling is Tenascin-C (TNC) [13]. TNC functions in structural signaling, influencing cell interactions with other ECM components [14]. In this context, the aim of the present study was to identify the immunohistochemical expression and distribution of Tenascin-C in OTGE patients.

## Materials and Methods

A descriptive observational study with non-probabilistic convenience sampling was conducted, comprising 65 subjects (30 women and 35 men). Two groups were established: The first consisted of periodontally healthy individuals not undergoing orthodontic treatment, indicated for crown lengthening for esthetic reasons (control, n=30); the second consisted of patients diagnosed with generalized OTGE (Seymour index ≥30), localized in the marginal and interdental gingiva, not exceeding one-third of the clinical crown (affected, n=35). These parameters were established according to Pinto *et al*. [4]. All participants underwent conventional periodontal treatment performed by a calibrated periodontist, consisting of bacterial biofilm control and ultrasonic and manual scaling. Inclusion criteria required an O'Leary plaque index ≤15%, systemic health, age over 18 years, and no pharmacological treatment. Smokers, pregnant women, and subjects who had undergone surgical interventions or periodontal treatment within the previous year were excluded. This study was reviewed and approved by the Research Ethics Committee of Corporación Universitaria Rafael Núñez (Code CE072023). Participants were informed of the study objectives and the purpose of sample processing and analysis. All participants completed, approved, and signed an informed consent form, prepared in accordance with the principles of the Declaration of Helsinki.

Surgical procedures were performed by a single periodontist under local anesthesia with 2% lidocaine and 1:80,000 epinephrine, administered via maxillary alveolar plexus blocks and mandibular vestibular infiltration, following external bevel and intrasulcular incisions with a No. 15c scalpel blade [15,16]. Patients were re-evaluated at 7 and 14 days after periodontal surgery; tissue healing was within expected parameters, with no complications, and patient satisfaction was confirmed.

*Tissue processing*

After gingival surgery, biopsies were fixed in 4% paraformaldehyde for 48 hours. Samples were then dehydrated in graded ethanol and xylene baths (Leica Biosystems, United States) for 5 minutes each. During paraffin embedding, biopsies were positioned parallel to the cutting plane to ensure subsequent observation of the gingival epithelial and connective tissue. Sections were cut using a microtome (Leica RM2125 RST, United States) at a thickness of 5 μm and mounted on polysine-coated adhesion slides (Thermo Scientific™, United States).

*Sirius Red staining*

Gingival tissue sections were deparaffinized with xylene and rehydrated in decreasing alcohol solutions, one 5-minute bath in each solution. Sections were then immersed in Mayer's hematoxylin for 5 minutes, followed by rinsing in running and distilled water. Sections were subsequently stained with Sirius Red dye for 15 minutes. Finally, sections were dehydrated in alcohol, washed in xylene, and mounted with DPX.

*Immunohistochemical analysis*

After deparaffinization with xylene (Leica Biosystems, United States), sections were rehydrated through graded alcohols. Tissue sections were placed in citrate buffer, pH 6, for 20 minutes at 97°C for antigen retrieval. To reduce nonspecific staining caused by endogenous peroxidase, sections were blocked with 3% hydrogen peroxide for 15 minutes at room temperature. Sections were then rinsed in wash solution (Tris-buffered saline (TBS 1X, Gibco™) + 0.05% Tween®20) for 5 minutes and incubated with normal horse serum (Vector Laboratories, United States) for 30 minutes. Incubation with the primary anti-Tenascin-C antibody (mouse monoclonal, 1:200 dilution, clone 4C8MS, Novus Biologicals, United States) was performed overnight at 4°C. The following day, tissue sections were incubated with a biotinylated anti-mouse secondary antibody (Vector Laboratories, United States) and with the R.T.U Vectastain® ABC system (Vector Laboratories, United States) for 30 minutes at room temperature. The reaction was visualized by incubating sections for 4 minutes with diaminobenzidine (ImmPACT DAB system, Vector Laboratories, United States). Finally, tissues were counterstained for 30 seconds with Mayer's hematoxylin, dehydrated in ethanol and xylene, and mounted using an anhydrous mounting medium (DPX, Sigma-Aldrich, United States).

The Tenascin-C immunohistochemical reaction was visualized as a brown color. It was evaluated semi-quantitatively in the gingival tissue according to immunohistochemical staining intensity. Tenascin-C intensity was classified as: 0 (absent), 1 (weak but detectable), 2 (moderate), and 3 (strong). All observations were performed by a calibrated pathologist using a microscope (Leica DM 500, Germany) coupled to a photographic camera; this primary observer was not masked to group allocation at the time of scoring. To assess reproducibility, all 65 sections were independently re-scored by a second observer, blinded to the primary scores and to group allocation.

*Statistical analysis*

Categorical distributions of Tenascin-C staining intensity between the control and OTGE groups were compared using the two-tailed Fisher's exact test for 2x2 comparisons and the chi-square test of independence for larger contingency tables; a *p* value <0.05 was considered statistically significant. Exact (Clopper-Pearson) 95% confidence intervals (CI) were calculated for the proportion of subjects showing a detectable basement membrane Tenascin-C band in each group (Table 1). For this outcome, all 30 control subjects showed a detectable band (100%; 95% CI 88.4-100%), compared with 5 of 35 OTGE patients (14.3%; 95% CI 4.8-30.3%); this between-group difference was statistically significant (Fisher's exact test, *p*<0.0001). The relative risk (RR) of a detectable basement membrane band in controls versus OTGE patients was 6.44 (95% CI 2.98-13.92); because the control group had a zero cell (0/30 undetectable), the RR and its CI were computed using the Haldane-Anscombe continuity correction (0.5 added to each cell). For blood vessel staining intensity (Table 2), the distribution differed significantly between groups (χ² (2, N=65) =40.86, *p*<0.0001; all expected cell counts ≥5). Inter-observer reproducibility of the semi-quantitative score was assessed on the full series of 65 sections, independently re-scored by a second, blinded observer; agreement was substantial (linear-weighted Cohen's kappa=0.79). Intra-observer reproducibility (repeat scoring by the same observer) was not assessed. No a priori sample size calculation was performed.

As a post-hoc precision assessment, the exact 95% confidence intervals obtained for the primary outcome (detectable basement membrane Tenascin-C band: 88.4-100% in controls vs. 4.8-30.3% in OTGE patients) were reasonably narrow and did not overlap, indicating that the achieved sample size provided adequate precision to detect this large between-group difference. This post-hoc assessment does not replace a prospective sample-size calculation and should not be interpreted as formal statistical power; estimates for secondary or more modest between-group differences may be less precise.

## Results

Participants had a mean age of 20.8 years (control group) and 23.2 years (affected group) (Table 3). Histological evaluation identified a keratinized stratified squamous epithelium in all control subjects. Keratinocytes showed good organization with no apparent modification of their anatomical features. The basal layer was composed of cuboidal cells with round or oval nuclei. A continuous basement membrane was observed between the epithelial and connective tissue. Sirius Red staining revealed the organization of epithelial tissue cells in the control group, with red collagen fiber bundles surrounding blood vessels and forming part of the basement membrane (Figure 1A). In OTGE patients, a hypertrophic and hyperplastic epithelium was identified, with keratinocyte nuclei showing a loss of organization. In the basal layer, keratinocytes did not maintain the parallel, continuous arrangement observed in control tissue samples. In all OTGE patients, Sirius Red staining revealed abundant collagen fibers distributed in dense bundles bordering blood vessels and cells present in the extracellular matrix. Collagen fibers in the basal lamina were intermingled with basal layer keratinocytes, and the clear boundary between epithelial and connective tissue observed in healthy subjects' samples was absent (Figure 1B).

Positive Tenascin-C staining was detected in all tissues from control group individuals. In these patients, Tenascin-C immunoreactivity was intensely observed as a delicate, continuous line in the area adjacent to and below the basal layer cells of the epithelial tissue (Figure 2A). Positive Tenascin-C immunostaining of blood vessels near epithelial projections was also observed in these tissues (Figure 3A,B). In the OTGE patient group, variable Tenascin-C immunoreactivity was observed. In 30 patients, the thin, continuous band of Tenascin-C was not detected as it was in the basement membrane area of control group participants. Only 5 patients showed a very delicate line, recorded as weak but detectable (Figure 2B). This difference in basement membrane Tenascin-C detection between groups was statistically significant (100%, 95% CI 88.4-100%, in controls vs. 14.3%, 95% CI 4.8-30.3%, in OTGE patients; Fisher's exact test, *p*<0.0001; Table 1). However, Tenascin-C immunolocalization was observed at the basal pole of keratinocytes located in the basal layer of the epithelial tissue in all OTGE patients (Figure 3C,D).

Tenascin-C was also concentrated in the endothelial cells forming blood vessels distributed in the gingival connective tissue of control group subjects, with moderate intensity in 10 subjects (33.3%) and strong intensity in 20 subjects (66.7%) (Figure 2C). In the OTGE group, weak immunohistochemical staining of blood vessels was identified in 20 patients (57.1%), while moderate staining was observed in 15 patients (42.9%) (Figure 2D). The overall distribution of blood vessel staining intensity differed significantly between groups (χ² (2, N=65) =40.86, *p*<0.0001; Table 2). Positive staining was also identified at the cytoplasmic level in gingival fibroblasts of all control group subjects. These cells were surrounded by collagen fibers (Figure 4A,B). Likewise, Tenascin-C immunolocalization was observed in the extracellular medium very close to gingival fibroblasts, which also showed positive staining (Figure 4C,D).

## Discussion

Tenascin-C belongs to the matricellular protein group; these proteins play prominent roles in mediating communication between cells and their surrounding extracellular environment. Given its essential role in regulating cell phenotype and tissue function, changes in its expression have been reported in response to injury, cellular stress, and in pathologies involving alterations in cell proliferation [17]. To our knowledge, this study is among the first to identify the immunohistochemical expression and distribution of Tenascin-C in OTGE patients.

Tenascin-C was present as a thin, continuous line along the basement membrane of periodontally healthy control group individuals. However, in OTGE patients, immunohistochemical expression was not observed at the interface between the epithelial and connective tissue. This distribution change is likely related to the loss of basement membrane continuity and increased plasticity of the gingival epithelium in OTGE patients. Increased tissue plasticity is observed in the process of epithelial-mesenchymal transition (EMT). We previously reported the presence of EMT-related markers, such as α-SMA and S100A4, in gingival tissues of OTGE patients [18,19]. EMT is regulated by a complex network of molecular mechanisms that induce morphological changes and phenotypic modifications in epithelial cells, which tend to acquire mesenchymal properties [20]. Studies show that both EMT and Tenascin-C are involved in tissue alterations related to excessive ECM accumulation, not only in pathologies affecting gingival tissue but also in several organs, including the kidneys, liver, and lungs [13,21].

The loss of balance among ECM components, as demonstrated by histological analysis in OTGE patients in this study, implies changes in tissue architecture. One such change could affect the arrangement of cellular elements and structures such as blood vessels. We estimate that mechanical tension generated by orthodontic forces, in addition to contributing to collagen accumulation, could be involved in an increase in blood vessel diameter, as previously noted in OTGE patients [18]. In the present study, in addition to confirming a significant number of blood vessels present in the gingival connective tissue of OTGE patients, a number of blood vessels with structural modifications were also observed, although these were not formally quantified. These structural changes did not preclude positive Tenascin-C immunohistochemical staining. Constitutive Tenascin-C expression in blood vessels has been related to elasticity and protection against mechanical stress [22]. However, the role it may play in hypertrophic gingival tissue remains to be established. Future studies will be necessary to understand the possible alteration in Tenascin-C immunohistochemical expression identified in the blood vessels of OTGE patients.

Histological observation revealed thickening of the epithelial tissue, accompanied by significant collagen fiber deposition in the basal lamina and deep connective tissue of OTGE patients, consistent with previously reported histological and cellular findings [23]. A significant increase in collagen IV, predominantly located in the basal lamina, was detected in orthodontic patients by Şurlin *et al*. [24]. These authors, who also investigated matrix metalloproteinase 9 (MMP-9), concluded that collagen IV increased in parallel with MMP-9, suggesting that inflammation and mechanical stress induced by orthodontic devices could be key to the development of the fibrotic process. Although the microscopic analysis performed in the present study did not reveal a chronic inflammatory infiltrate, perhaps because patients with an acceptable periodontal health status were included, we do not rule out that inflammation caused by poor oral hygiene, together with increased periodontal bacteria and proinflammatory cytokines, as confirmed by our team and other studies, may be responsible for OTGE [7,25].

It is likely that the increase in collagen observed in the orthodontic patients analyzed in this study results from the effect of mechanical forces on ECM proliferation and synthesis. It is possible that dysregulation of the homeostasis between collagen production and its inhibition by enzymes such as MMPs may contribute to hypertrophic gingival tissue, as observed in the orthodontic patients analyzed in this study and reported in previous work by our team [26]. Results reported by Nan *et al*. [27], who cultured gingival fibroblasts on 3D scaffolds and subjected them to different mechanical forces, revealed a significant increase in cell proliferation and type I collagen expression, along with a decrease in MMP-1. Interestingly, these authors reported a decrease in the effects of mechanical forces on gingival fibroblast proliferation, collagen I, and MMP-1 when a transforming growth factor beta 1 (TGF-β1) inhibitor was applied. Our group has preliminary, not-yet-peer-reviewed observations suggestive of increased TGF-β1 immunohistochemical expression in gingival tissues of OTGE patients; these are mentioned here only as a rationale for future hypothesis-driven work and should not be regarded as evidence supporting the mediator role proposed for TGF-β1 in this process until they have been independently verified and published.

Differentiation of fibroblasts into collagen-producing myofibroblasts can be stimulated by TGF-β1. A study by Kaarteenaho-Wiik *et al*. [28], who studied experimentally induced differentiation of fibroblasts into myofibroblasts, found that after 6 hours of TGF-β1 exposure, cells were positive for α-SMA and extracellular elements such as fibronectin and Tenascin-C. Immunolocalization of α-SMA in gingival fibroblasts (presumably myofibroblasts) in hypertrophic gingival tissue of orthodontic patients has been previously reported by us [19]. Here, we have shown Tenascin-C immunodetection at the extracellular level near fibroblastic cells in OTGE patients. It is likely that TGF-β1 involvement would be crucial in the fibrotic process observed in OTGE patients and would induce functional and spatial modifications of Tenascin-C. The molecular details of these processes should be studied in future research.

Another potential triggering factor for OTGE in patients with adequate oral hygiene is the cytotoxic effect induced by orthodontic bands. Our team reported significantly higher levels of nickel bioaccumulation in saliva, bacterial biofilm, and gingival tissue in OTGE patients compared to samples from patients without gingival enlargement [10]. These results suggest that high nickel concentrations may be related to OTGE. Recently, Bandeira *et al*. [29] evaluated the toxicity and oxidative stress response induced by orthodontic bands on human gingival fibroblasts. Results indicated cytotoxicity and induction of antioxidant gene response in gingival fibroblasts. The role of oxidative stress in OTGE pathogenesis has been scarcely addressed, but analyses conducted in individuals affected by hereditary gingival enlargement suggest that oxidative stress may influence collagen production and could play a crucial role in OTGE pathogenesis [30].

The interpretation of these findings should also take into account the non-probabilistic, convenience-based recruitment strategy used in this study, as well as the choice of a control group composed of individuals referred for esthetic crown lengthening rather than a randomly sampled periodontally healthy population. Both features may have introduced selection bias related to gingival biotype, baseline extracellular matrix turnover, or other unmeasured characteristics of the control group, and could limit the extent to which the observed differences in Tenascin-C distribution can be generalized to the broader population of orthodontic patients. Because the primary observer was also not masked to group allocation during the initial semi-quantitative scoring, this selection bias could in principle interact with observer-related bias to influence the magnitude of the reported associations; the substantial inter-observer agreement (weighted kappa=0.79) obtained with a second, blinded observer partially mitigates but does not eliminate this concern.

The results presented here contribute to a growing understanding of how orthodontic treatment affects gingival tissue components, particularly Tenascin-C. It is worth noting that OTGE patients show changes in the expression and distribution of Tenascin-C in the basement membrane, fibroblasts, and blood vessels of the gingival connective tissue. Future research will be valuable in elucidating the pathophysiological mechanisms of Tenascin-C in gingival enlargement. Such analyses would be needed before Tenascin-C could be considered a candidate molecular marker of extracellular matrix remodeling in fibrotic processes such as OTGE; its potential diagnostic or therapeutic value remains speculative at this stage and would require validation through diagnostic-performance analyses and longitudinal studies.

This study has several limitations that should be considered when interpreting the findings. First, the control group consisted of individuals referred for esthetic crown lengthening rather than a random sample of periodontally healthy, non-orthodontic individuals; this selection strategy may not be representative of the general population and could introduce selection bias related to gingival biotype or extracellular matrix turnover. Second, the sample was obtained by non-probabilistic convenience sampling without an a priori sample size calculation, which limits the precision and generalizability of the estimates. Third, Tenascin-C staining intensity was scored semi-quantitatively (0-3) by a single primary observer who was not masked to group allocation; however, inter-observer reproducibility, assessed on the full series of 65 sections re-scored by a second, blinded observer, was substantial (linear-weighted kappa=0.79), which supports the reliability of the semi-quantitative grading. Intra-observer reproducibility was not assessed, and future studies would still benefit from complementing visual grading with digital image analysis (optical density quantification). Fourth, potential confounders such as the age difference between groups, orthodontic appliance type (fixed vs. removable), and duration of treatment were not controlled for in the analysis; in particular, standard deviations for age were not available, precluding a formal statistical comparison of this potential confounder. Finally, the observational, cross-sectional design does not permit causal inference regarding the relationship between orthodontic mechanical forces and the observed changes in Tenascin-C expression; the mechanistic interpretations offered in the Discussion should be regarded as hypothesis-generating rather than confirmatory.

## Conclusions

Patients with orthodontic treatment-induced gingival enlargement exhibited marked differences in the immunohistochemical expression and distribution of Tenascin-C compared with periodontally healthy controls. In OTGE specimens, the characteristic continuous Tenascin-C band at the epithelial-connective tissue interface was absent in most cases, and differences in Tenascin-C localization within gingival fibroblasts and blood vessels were observed alongside epithelial disorganization and abundant collagen deposition. These findings demonstrate an association between OTGE and altered Tenascin-C distribution within gingival tissues. Given the observational, cross-sectional design of this study, no conclusions can be drawn regarding causality, diagnostic or prognostic performance, or therapeutic utility.

## Figures and Tables

**Table 1:** Table 1: Distribution of Tenascin-C immunostaining intensity in the gingival basement membrane, by group.

**Score**	**Description**	**Control, n (%)**	**OTGE, n (%)**
0	Absent	0 (0.0)	30 (85.7)
1	Weak but detectable	0 (0.0)	5 (14.3)
2	Moderate	0 (0.0)	0 (0.0)
3	Strong	30 (100.0)	0 (0.0)
Total		30 (100.0)	35 (100.0)

Detectable band (score ≥1) vs. undetectable (score 0), control vs. OTGE: Fisher's exact test, p<0.0001. Relative risk of a detectable basement membrane band, control vs. OTGE: 6.44 (95% CI 2.98-13.92; Haldane-Anscombe continuity-corrected estimate, applied because of a zero cell in the control group).

**Table 2:** Table 2: Distribution of Tenascin-C immunostaining intensity in gingival blood vessels, by group.

**Score**	**Description**	**Control, n (%)**	**OTGE, n (%)**
0	Absent	0 (0.0)	0 (0.0)
1	Weak but detectable	0 (0.0)	20 (57.1)
2	Moderate	10 (33.3)	15 (42.9)
3	Strong	20 (66.7)	0 (0.0)
Total		30 (100.0)	35 (100.0)

Chi-square test of independence (2x3 table restricted to non-empty categories: Weak, moderate, strong): χ^2^ (2, N=65) =40.86, p<0.0001. All expected cell counts were ≥5, satisfying the assumptions of the test.

**Table 3:** Table 3: Demographic characteristics of study participants.

**Characteristic**	**Control (n=30)**	**OTGE (n=35)**
Mean age, years	20.8	23.2
Gender (women/men), total sample*	30/35 (overall)	30/35 (overall)

*Gender distribution (30 women, 35 men) was reported for the total sample only in the source data and could not be disaggregated by group; standard deviations for age were not available and therefore a formal between-group comparison of age was not performed (see Limitations).

**Figure 1:**
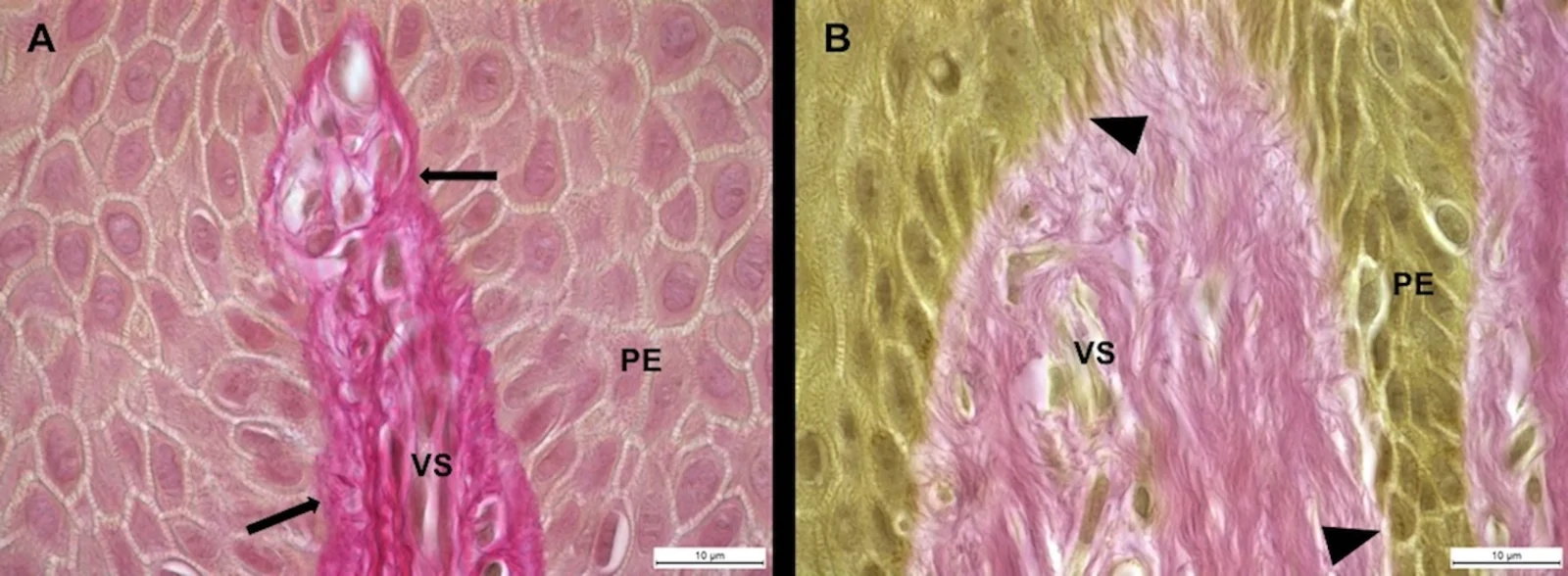
Fig. 1: Sirius Red staining in gingival tissue. A: Identification of organized basement membrane keratinocytes. Presence of collagen fibers delimiting the epithelial and connective tissue (arrows) in a control group individual. B: Irregularly arranged keratinocytes surrounded by abundant collagen fibers (triangles) in an orthodontic patient with gingival enlargement. PE: Epithelial projection; VS: Blood vessel; black bar: 10 µm.

**Figure 2:**
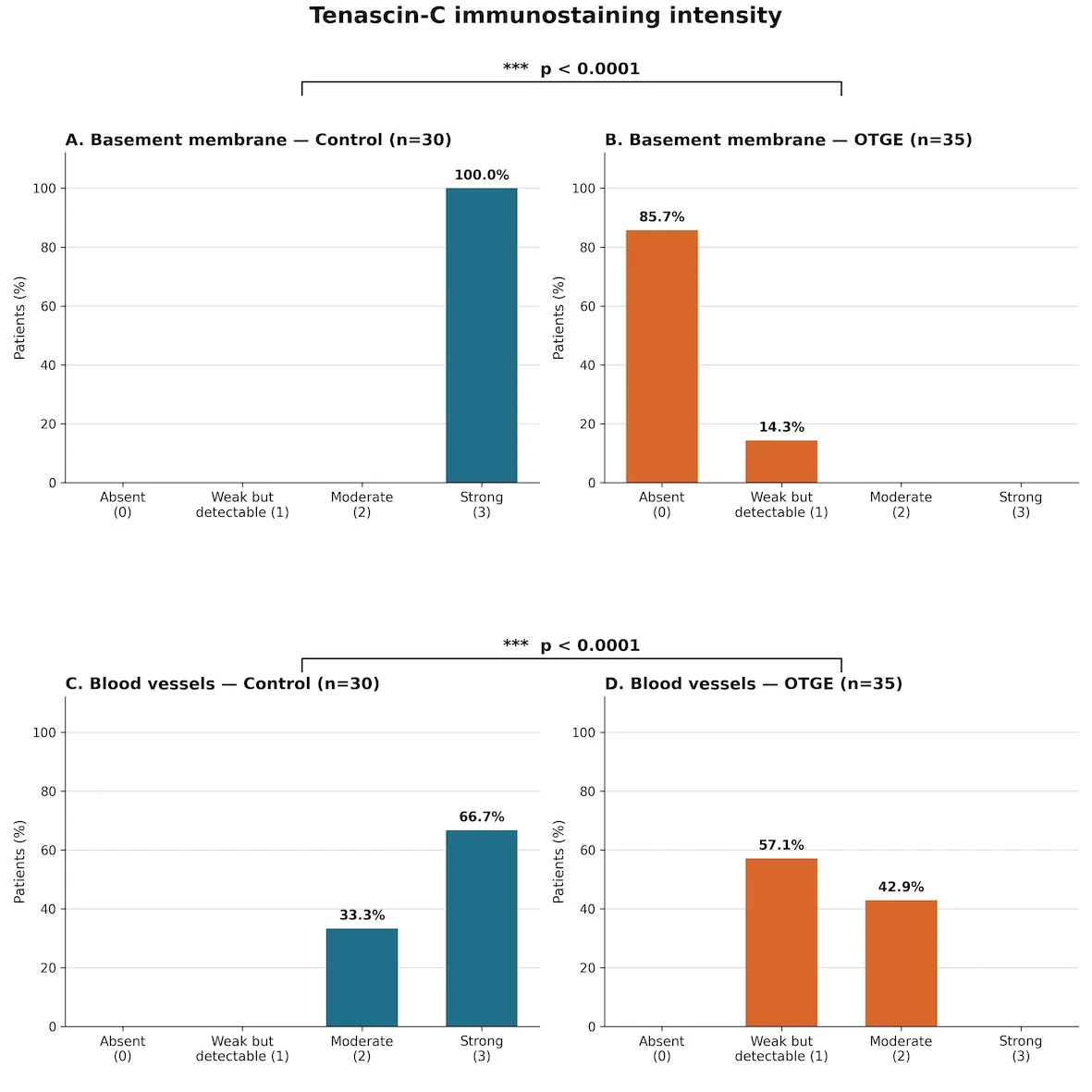
Fig. 2: Percentage of Tenascin-C immunostaining intensity, by group. A: Basement membrane, control group. B: Basement membrane, OTGE group. C: Blood vessels, control group. D: Blood vessels, OTGE group. ***p<0.0001 vs. control (A vs. B: Fisher's exact test; C vs. D: Chi-square test of independence).

**Figure 3:**
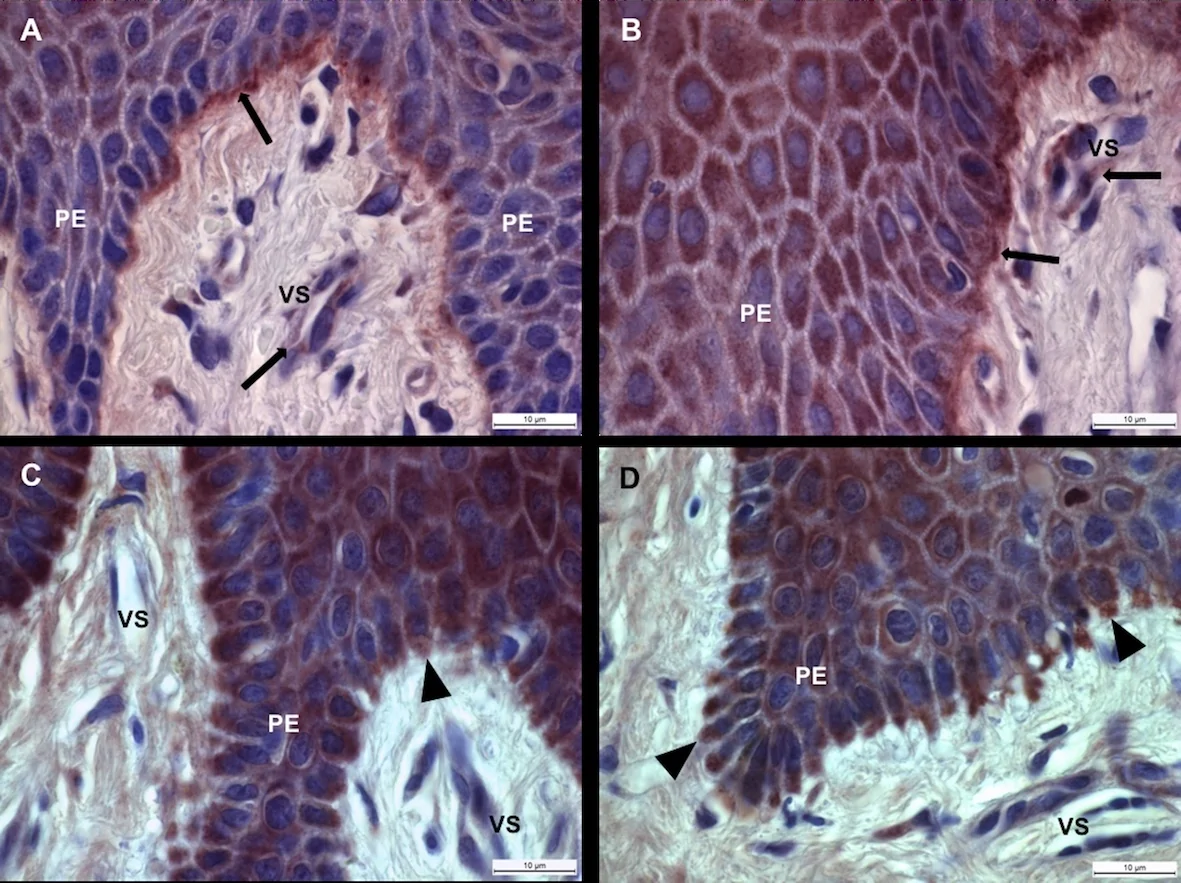
Fig. 3: Tenascin-C immunolocalization in the basal lamina and blood vessels (arrow) of a healthy donor (A,B) and an orthodontic patient with gingival enlargement (triangle) (C,D). PE: Epithelial projection; VS: Blood vessel; black bar: 10 µm.

**Figure 4:**
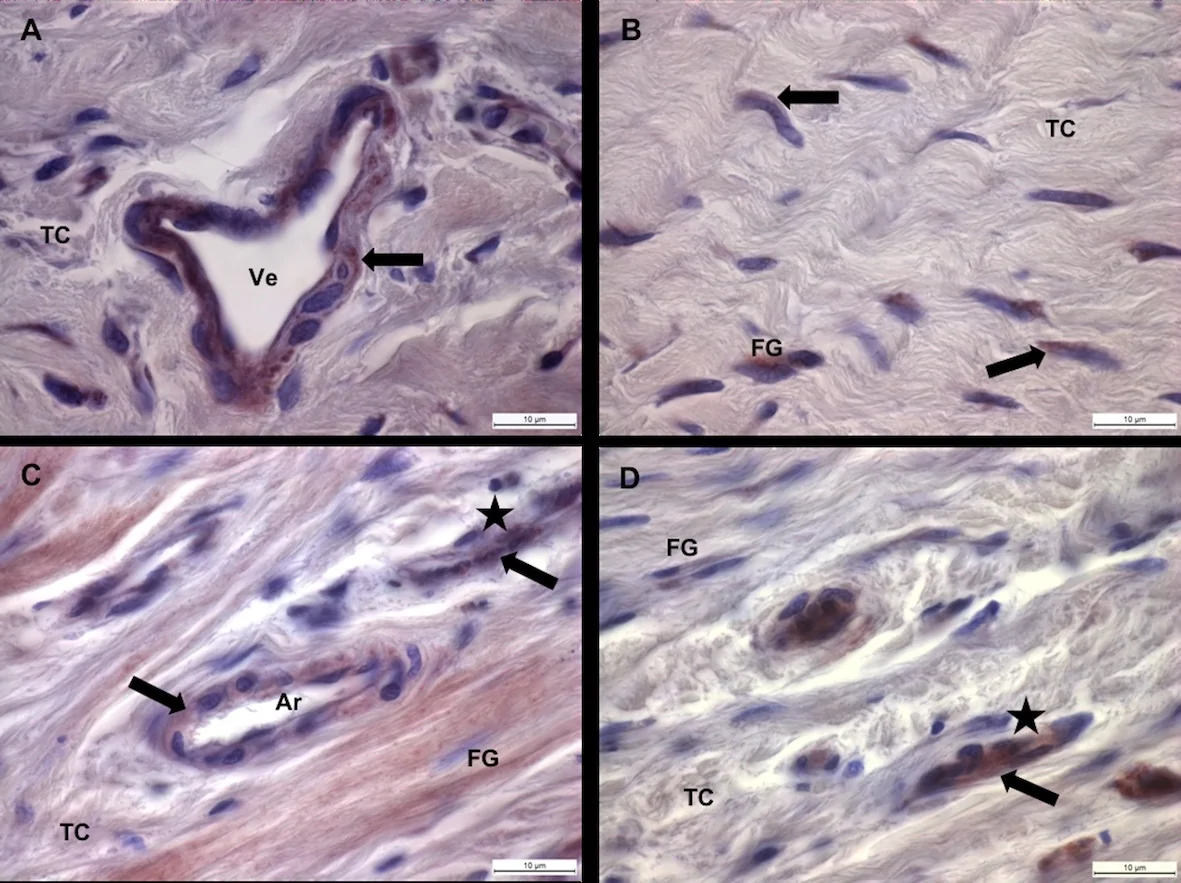
Fig. 4: Tenascin-C immunohistochemical detection in blood vessels and gingival fibroblasts (FG) (arrow) of a healthy donor (A,B) and a patient with orthodontic-associated gingival enlargement (C,D). Note the Tenascin-C immunostaining in the extracellular medium near FG in patients with gingival enlargement. Star: Tenascin-C positive in blood vessels. TC: Connective tissue; Ar: Arteriole; Ve: Venule; FG: Gingival fibroblast; black bar: 10 µm.

## Data Availability

All data generated or analyzed during this study are included in this published article.
